# Temporal changes in physiological parameters in Chinese adults during a month-long 5:2 fasting regimen: an exploratory observational study

**DOI:** 10.3389/fnut.2026.1866854

**Published:** 2026-09-07

**Authors:** Yingying Zhuang, Hongfei Mi, Xinyue Wang, Sheng Jia, Chengwei Huang, Nishang Zheng, Siyi Wang, Jian Gao, Chenghao Su

**Affiliations:** 1 Department of Clinical Nutrition, Zhongshan Hospital (Xiamen), Fudan University, Xiamen, China; 2 Xiamen Clinical Research Center for Cancer Therapy, Xiamen, China; 3 Xiamen Key Laboratory of Biotherapy, Xiamen, China; 4 Department of Public Health, Zhongshan Hospital (Xiamen), Fudan University, Xiamen, China; 5 Department of Information, Zhongshan Hospital (Xiamen), Fudan University, Xiamen, China; 6 Department of Nutrition, Zhongshan Hospital, Fudan University, Shanghai, China

**Keywords:** 5:2 fasting regimen, obesity, physiological parameters, population, sex-specific

## Abstract

**Background:**

The 5:2 intermittent fasting regimen (5 days of unrestricted eating + 2 days of strict calorie restriction) has been widely adopted as a weight management strategy. However, the short-term temporal changes in body composition and its sex-specific effects on body composition remain unclear. Particularly, the relationship between weight loss and lean body mass during the weight reduction process has not been sufficiently studied.

**Methods:**

This retrospective cohort analyzed serial body composition data from 109 overweight/obese adults (28 Men/81 Female; age 31.4 ± 7.5 vs. 34.4 ± 9.9, *p* = 0.193) undergoing 4-week 5:2 fasting with weekly monitoring. Exclusions: age <18y or ≥60y, treatment <4 weeks, or irregular follow-up.

**Results:**

Both sexes exhibited linear reductions in weight, body fat percentage, and visceral adipose tissue (*p* < 0.05). A modest decline in lean mass occurred, indicating that muscle preservation deserves attention in future studies. Weight loss correlated with baseline muscle mass (*r*^2^ = 0.060, *p* = 0.027) and basal metabolic rate (BMR; *r*^2^ = 0.059, *p* = 0.029) in female, whereas no such associations were found in males (*p* > 0.05). Age showed no significant association with outcomes (*p* > 0.05).

**Conclusion:**

The 5:2 regimen induces controlled multi-parameter improvements in adiposity metrics, with a concomitant trend toward lean mass reduction that merits attention. Baseline muscle mass and BMR were significant correlates of weight loss in females, suggesting that muscle-centric strategies may be particularly relevant for this subgroup. In males, weight loss appeared to be primarily associated with energy intake regulation rather than baseline body composition. These findings are observational and hypothesis-generating; causality cannot be inferred. Future prospective studies are warranted to validate these sex-specific associations.

## 1 Introduction

The global prevalence of obesity continues to escalate. According to 2022 data, over 1 billion individuals worldwide are classified as obese, representing 13% of the global population. Obesity constitutes a major public health challenge, posing a severe threat to individual health and imposing a substantial socioeconomic burden (1, 2). China faces an equally serious situation: data from the China Center for Disease Control and Prevention (2015–2019) indicate that overweight and obesity rates among Chinese adults aged 18 and above reached 34.3 and 16.4%, respectively, with both showing persistent upward trends (3, 4). Dietary intervention serves as the cornerstone of obesity treatment, with its core objective being the achievement of sustained energy deficit – where energy intake consistently falls below expenditure – thereby forcing the body to utilize stored fat for energy and facilitating weight (5–7).

Traditional continuous caloric restriction, while effective in reducing body weight and improving certain metabolic markers (8, 9), exhibits significant limitations in clinical application: poor long-term adherence and frequent, substantial loss of lean mass (10, 11). Lean mass loss not only reduces basal metabolic rate, hindering long-term weight maintenance, but may also impair muscle function and overall health (12). Intermittent energy restriction, often implemented via intermittent fasting regimens like the 5:2 approach (5 days of normal eating and 2 days of very lower calorie intake: 500–600 kcal per week), centers on periodic energy intake modulation. Intermittent energy restriction is proposed to induce “metabolic switching,” offering potential advantages such as promoting fat oxidation, enhancing insulin sensitivity, and activating cellular autophagy (13–15). Its non-continuous nature may also improve adherence (16). Clinical studies confirm that the 5:2 fasting regimen achieves significant weight loss and improvements in lipid and glucose profiles, demonstrating effects comparable to or even superior to CR (17–20). Two recent meta-analyses have further consolidated these findings, showing that while the 5:2 diet effectively reduces body weight, BMI, waist circumference, and fat mass, it is also associated with a modest but statistically significant reduction in fat-free mass—of which skeletal muscle is the primary component (13, 21). Thus, although the 5:2 regimen is efficacious for weight management, the accompanying lean tissue loss warrants close attention.

However, the evidence base for this concern remains incomplete in three important respects. First, the above meta-analyses were based on pre- and post-intervention measurements, which cannot capture when or how rapidly lean mass loss occurs during the intervention (22). Whether the majority of muscle loss happens within the first 1–2 weeks—a period when weight reduction is confounded by fluid and glycogen shifts—or progresses linearly over time, has not been resolved under high-frequency monitoring. Second, group-averaged effect sizes obscure considerable individual heterogeneity (23); it remains unknown whether baseline muscle mass or fat mass predicts the magnitude of lean tissue loss, and thus whether certain subgroups are disproportionately affected. Third, sex-specific response patterns remain inconclusive. While some studies have reported that women may experience greater reductions in lean mass during intermittent fasting (24, 25), others have observed the opposite—that obesity and/or high insulin levels promote lean mass loss during an acute fast in men rather than in women (22). Still other investigations have reported no significant sex differences in lean mass changes (26). Consequently, while the phenomenon of lean mass reduction is well documented, the temporal dynamics, individual determinants, and sex-specific patterns of this process remain uncharted.

To explore these gaps, the present study employs a weekly repeated-measures design using bioelectrical impedance analysis over a 4-week intervention period. This monitoring frequency allows for the characterization of body composition trajectories during the acute phase of the 5:2 dietary intervention. In addition, we perform sex-stratified analyses and examine interactions between baseline body composition and subsequent changes, with the goal of exploring whether baseline physiological status and sex may be associated with differential responses to the regimen. Through this descriptive approach, we hope to offer preliminary observations that could help generate hypotheses for refining the 5:2 fasting protocol, particularly regarding the potential need for muscle-preserving strategies and whether such strategies might differ between males and females.

## 2 Materials and methods

### 2.1 Data source and study sample

This was a retrospective analysis of prospectively collected clinical intervention data from a 4-week weight loss program. Electronic database records of overweight or obese participants who underwent a 4-week weight loss intervention at Zhongshan Hospital (Xiamen), Fudan University between January 1, 2020, and January 1, 2024, were reviewed.

Inclusion criteria were: (1) age 18–60 years; (2) BMI ≥ 24 kg/m^2^; (3) completion of the 4-week weight loss intervention; and (4) fixed weekly follow-up visits. Participants were excluded if they could not complete the 4-week intervention. The study protocol was approved by the Ethics Committee of Zhongshan Hospital, Fudan University (Approval No. B2024-065R2).

### 2.2 Dietary intervention

On fasting days (two non-consecutive days per week), participants were instructed to consume approximately 500–600 kcal/day, corresponding to approximately 30% of their baseline BMR measured by the H-Key350 device. On non-fasting days, participants were advised to consume an amount equivalent to their full measured BMR (100%), with a targeted macronutrient composition of 50–60% from carbohydrates, 25–30% from fat, and 10–15% from protein. On fasting days, the recommended macronutrient composition was 20–25% from protein, 25–30% from fat, and 45–55% from carbohydrates, emphasizing high-quality protein sources (e.g., lean meat, fish, eggs, and legumes) to support satiety and lean mass preservation. This measured BMR served as the individualized reference for prescribing calorie targets on both fasting and non-fasting days. No nutritional supplements were prescribed or systematically used. Participants were advised to obtain all nutrients from whole foods. Adherence to the dietary prescription was not quantitatively assessed using structured dietary records; rather, adherence was inferred from attendance at weekly follow-up visits, where weight and body composition changes were monitored and participants received dietary counseling. Similarly, adherence to exercise recommendations was not objectively monitored, and quantitative data on actual physical activity levels were not collected. Participants were instructed to complete 150–300 min per week of moderate-intensity aerobic or resistance exercise. Several behavioral strategies were recommended, including: reducing intake of high-fat foods, maintaining regular meal times, consuming approximately five meals daily, adopting a specific eating sequence (vegetables and protein first, followed by carbohydrates), increasing the number of chewing cycles per bite, daily self-weighing, avoiding prolonged sedentary periods, engaging in daily physical activity, and maintaining an early sleep schedule. Weight and body composition data were collected during weekly clinical visits. Dietary education was provided by trained registered dietitians or clinicians.

### 2.3 Measures

#### 2.3.1 Outcome measures

The primary outcome measures were changes in body weight, body fat percentage (BFR), and visceral fat area (VFA) over the 4-week intervention period. Secondary outcomes included changes in lean mass, basal metabolic rate, waist circumference, hip circumference, waist-to-hip (WHR), and segmental body composition (fat and lean mass of the upper limbs, trunk, and lower limbs).

#### 2.3.2 Body composition assessment

The H-Key 350 device (Beijing Sihai Huachen Biotechnology Co., Ltd., China) is reported by the manufacturer to have been validated against DXA, with correlation coefficients of r > 0.90 for fat and lean mass estimates (manufacturer’s internal data). After an 8-h overnight fast, body composition was measured at baseline (0 weeks) and at 1, 2, and 4 weeks. All measurements were performed with participants voiding their bladder immediately before assessment, in a standardized supine position, at the same time of day (8:00–10:00 a.m.) to minimize circadian and hydration-related variations. Participants were instructed to maintain habitual fluid intake and avoid strenuous exercise 24 h prior to each measurement. The device output included the following parameters:

Anthropometrics: Weight, BMI, Waist Circumference (Waist), Hip Circumference (Hip), Waist-to-Hip Ratio (WHR).

Energy Metabolism: Basal Metabolic Rate (BMR).

Fat Mass Indices: Body Fat Percentage (BFR), Visceral Fat Area (VFA).

Segmental Fat Mass:

Absolute Mass (kg): Left Upper Limb Body Fat (LUL-BF), Right Upper Limb Body Fat (RUL-BF), Trunk Body Fat (T-BF), Left Lower Limb Body Fat (LLL-BF), Right Lower Limb Body Fat (RLL-BF).

Ratio to Normal Reference (%): LUL/Normal-BF ratio %, RUL/Normal-BF ratio %, T/Normal-BF ratio %, LLL/Normal-BF ratio %, RLL/Normal-BF ratio %.

Lean Mass Indices:

Absolute Mass (kg): Total Lean Mass (Lean Mass), Left Upper Limb Mass (LUL-M), Right Upper Limb Mass (RUL-M), Trunk Mass (T-M), Left Lower Limb Mass (LLL-M), Right Lower Limb Mass (RLL-M). (Corrected typo from “LLL-BF” to “LLL-M”).

Ratio to Normal Reference (%): LUL/Normal-M ratio %, RUL/Normal-M ratio %, T/Normal-M ratio %, LLL/Normal-M ratio %, RLL/Normal-M ratio %.

Other Body Components: Protein Mass, Mineral Mass (referred to as “Isalt,” likely meaning inorganic salts/minerals).

Body Water Composition: Total Body Water (TBW), Extracellular Water (ECW), Intracellular Water (ICW).

Edema/Volume Indices: Edema Index (ECW-based) (EI-ECW), Edema Index (ECF-based) (EI-ECF). (Assuming ECF refers to Extracellular Fluid).

Derived Indices: Obesity Degree (calculated as: Body Weight / Target Weight * 100%), Muscle Control, Fat Control, Body Weight Control. (Specific definitions for “Control” indices would ideally be provided).

For the purposes of this exploratory analysis, we grouped the broad panel of BIA-derived parameters into five categories: (1) body morphology (weight, BMI, waist circumference, hip circumference, and WHR); (2) body water and solid composition balance (total body water, extracellular water, intracellular water, protein, and mineral); (3) total and segmental fat mass, expressed as both absolute mass (kg) and ratio to normative reference (%); (4) total and segmental lean/muscle mass, similarly expressed as absolute mass and ratio to normative reference; and (5) derived control indices (obesity degree, muscle control, fat control, body weight control, and edema indices).

### 2.4 Data collection

Clinical data, including age, sex, weight, and body composition parameters measured at 0, 1, 2, and 4 weeks, were collected from the independent electronic database. Data extraction and entry were performed independently by two trained researchers to ensure consistency and completeness. Discrepancies in information were resolved through consensus or by retrieving additional information from the electronic database.

### 2.5 Statistical methods

No *a priori* sample size calculation was performed, consistent with the retrospective and exploratory nature of this investigation. The sample size was determined by the availability of eligible participants who met the inclusion criteria during the recruitment period (January 2020 to January 2024). All analyses should be considered exploratory and hypothesis-generating. All statistical analyses were performed in R (version 4.3.0), with continuous variables presented as group-wise means ± standard deviations (SD) for baseline comparisons, and as mean ± SD at baseline and week 4 for each sex in the pre-to-post intervention analyses. A two-stage hypothesis testing framework was implemented beginning with assumption verification using Shapiro–Wilk tests for normality (*p* > 0.05 required per group) and Levene’s tests for homogeneity of variance (*p* > 0.05 required), followed by either one-way ANOVA with Tukey HSD post-hoc analysis when both assumptions were satisfied or Kruskal-Wallis tests with Bonferroni-adjusted Dunn’s post-hoc when assumptions were violated, using a significance threshold of *α* = 0.05. For baseline sex comparisons, effect sizes were quantified as Cohen’s d, calculated as the mean difference divided by the pooled standard deviation. To evaluate pre-to-post intervention changes within each sex, paired t-tests or Wilcoxon signed-rank tests were used depending on the normality of the difference scores. For each outcome, the mean change (post-pre) and its 95% confidence interval (CI) were calculated to quantify the magnitude and precision of the change. To address the issue of multiple comparisons across the large number of outcome variables, *p* values were adjusted using the Benjamini–Hochberg false discovery rate (FDR) method, and adjusted *p* values (*P*_fdr) are reported alongside the raw *p* values. Effect sizes for paired comparisons were quantified as Cohen’s dz., calculated as the mean difference divided by the standard deviation of the difference scores. According to Cohen’s conventions, |dz.| values of 0.2, 0.5, and 0.8 were interpreted as small, medium, and large effects, respectively. To analyze sex-specific temporal trends in anthropometric measures, we employed grouped trajectory visualization featuring non-parametric LOESS smoothing with data-adaptive bandwidth and tricube kernel weighting, incorporating both individual trajectories and group-level trend lines. Longitudinal dynamics and sex differences were quantified through linear mixed-effects models with fixed effects for time, sex, and baseline age, along with random intercepts and slopes to account for individual heterogeneity, using an unstructured variance–covariance matrix. Linear mixed-effects models included random intercepts and slopes for each participant. The random intercept accounts for individual differences in baseline values, including baseline BMI and other anthropometric measures, thereby adjusting for inter-individual heterogeneity without introducing collinearity with fixed effects. Change values (*Δ*) were computed for all anthropometric variables as Δ = Yfollow-up - Ybaseline, with pairwise linear associations between Δ-metrics evaluated via Pearson correlation analysis. Finally, weight-loss subphenotypes were identified through unsupervised K-means clustering of multidimensional Δ-metrics, where optimal cluster numbers were determined using silhouette width and elbow methods, with subtypes defined by centroid profiles. To evaluate the stability of the resulting cluster solutions, we performed internal validation via Bootstrap resampling with 1,000 iterations. Cluster stability was quantified using the Jaccard similarity index, with values > 0.85 considered highly stable, 0.70–0.85 considered moderately stable, and < 0.60 considered unstable. The Bootstrap validation results are summarized in Supplementary Table S3.

## 3 Results

### 3.1 Participants’ characteristics by sex

From January 1, 2020, to January 1, 2024, 652 overweight or obese individuals sought weight loss treatment at the same nutrition clinic. A total of 235 participants were excluded due to age <18 years or >60 years, and 256 were excluded for receiving treatment for less than 4 weeks. An additional 52 participants were excluded for not adhering to the weekly follow-up schedule. Consequently, 109 overweight or obese patients meeting the inclusion criteria were enrolled. To evaluate the potential impact of selection bias, we compared the baseline characteristics of the final included participants (n = 109) with those of the screened population (n = 417)- defined as individuals who met the initial age criteria (18–60 years) after excluding the 235 participants outside this age range from the initial 652. As summarized in Supplementary Table S1, no significant differences were observed between the two groups in age, sex distribution, BMI, body fat percentage, or lean mass (all *P*_fdr > 0.05), suggesting that the final included sample was broadly representative of the age-eligible overweight/obese population seeking treatment at our center.

The baseline characteristics of the finally participants are presented in Table 1. Among the participants, 28 were male and 81 were female. The mean ages were 31.39 ± 7.52 years and 34.44 ± 9.87 years, respectively (*p* = 0.193), indicating no statistically significant difference. However, males presented with significantly higher initial mean body weight, BMI, BMR, waist circumference, hip circumference, WHR, BFR, VFA, TBW, ECW, ICW, EI-ECW, EI-ECF, EI-ECF, and protein levels compared to females. Although fat mass in the upper limbs and trunk, as well as the ratio to normal values, were higher in males than in females (all *p* < 0.05), lower limb fat mass showed no significant sex-based difference. Notably, muscle mass in the limbs and trunk was higher in males than in females. However, the ratio of limb muscle mass to normal values showed no significant sex difference, and lower limb muscle mass was below normal values in both sexes. Obesity degree, calculated based on initial body weight, was higher in males. Both sexes showed the need for an increase in muscle mass, with no significant difference in the magnitude of this need between males and females. While both weight and body fat warranted reduction, the amount needing control was higher in males.

**Table 1:** Sex-specific baseline characteristics of the study participants.

Characteristics	Male	Female	*p-value (FDR)*	*Cohen’s dz*
*N*	28	81		
Age	31.39 ± 7.52	34.44 ± 9.87	0.222	−0.327
Weight (kg)	102.89 ± 20.28	75.40 ± 11.20	<0.001	1.956
BMI (kg/m^2^)	33.21 ± 5.51	28.93 ± 3.79	<0.001	0.998
BMR (kcal)	1787.54 ± 183.66	1350.07 ± 104.45	<0.001	3.388
Waist (cm)	114.46 ± 16.94	96.25 ± 9.56	<0.001	1.535
Hip (cm)	112.40 ± 8.74	102.63 ± 6.28	<0.001	1.4
WHR	1.01 ± 0.08	0.94 ± 0.05	<0.001	1.146
BFR (%)	35.27 ± 6.12	39.31 ± 4.87	<0.001	−0.776
VFA (cm^2^)	166.84 ± 60.13	145.98 ± 37.77	0.136	0.469
LUL-BF	3.49 ± 2.24	2.46 ± 0.93	0.022	0.743
LUL/Normal-BF ratio %	562.47 ± 339.18	267.23 ± 100.03	<0.001	1.545
RUL-BF	3.46 ± 2.25	2.44 ± 0.94	0.026	0.735
RUL/Normal BF ratio %	558.07 ± 340.44	264.66 ± 100.44	<0.001	1.53
T-BF	19.05 ± 5.87	15.03 ± 3.50	<0.001	0.95
T/Normal BF ratio %	441.02 ± 126.68	292.07 ± 65.28	<0.001	1.751
LLL-BF	4.86 ± 1.48	4.41 ± 1.17	0.136	0.362
LLL/Normal BF ratio %	276.05 ± 76.85	188.50 ± 49.71	<0.001	1.515
RLL-BF	4.92 ± 1.51	4.43 ± 1.18	0.127	0.388
RLL/Normal BF ratio %	279.31 ± 78.17	189.66 ± 50.37	<0.001	1.529
Lean mass	61.84 ± 7.99	42.67 ± 4.55	<0.001	3.41
LUL-M	3.80 ± 0.66	2.35 ± 0.37	<0.001	3.15
LUL/Normal M ratio %	103.09 ± 10.57	105.77 ± 12.10	0.588	−0.229
RUL-M	3.83 ± 0.62	2.39 ± 0.36	<0.001	3.282
RUL/Normal M ratio %	103.75 ± 10.66	107.10 ± 12.44	0.588	−0.279
T-M	29.41 ± 3.84	20.58 ± 2.25	<0.001	3.223
T/Normal M ratio %	100.37 ± 6.54	102.44 ± 5.73	0.359	−0.348
LLL-M	10.03 ± 1.35	6.87 ± 0.87	<0.001	3.142
LLL/Normal M ratio %	97.76 ± 5.41	97.47 ± 5.20	0.820	0.055
RLL-M	10.03 ± 1.31	6.89 ± 0.87	<0.001	3.142
RLL/Normal M ratio %	97.81 ± 5.61	97.65 ± 5.36	0.889	0.031
TBW	48.08 ± 6.21	33.21 ± 3.53	<0.001	3.403
ECW	18.06 ± 2.38	12.59 ± 1.34	<0.001	3.287
ICW	30.00 ± 3.86	20.62 ± 2.21	<0.001	3.448
EI-ECW	0.38 ± 0.00	0.38 ± 0.00	0.002	−0.74
EI-ECF	0.33 ± 0.00	0.33 ± 0.00	0.003	−0.698
Isalt	4.58 ± 0.65	3.25 ± 0.37	<0.001	2.909
Protein	12.97 ± 1.68	8.91 ± 0.95	<0.001	3.445
Obesity Degree	150.94 ± 25.02	137.76 ± 18.03	0.004	0.658
Muscle Control (kg)	0.38 ± 1.37	0.27 ± 0.67	0.525	0.125
Fat Control (kg)	−25.61 ± 12.43	−16.39 ± 7.06	<0.001	−1.055
Body Weight Control (kg)	−25.23 ± 12.96	−16.12 ± 7.31	<0.001	−1.055

*P*-value (FDR) indicates P values adjusted for multiple comparisons using the Benjamini–Hochberg false discovery rate method. Effect size is Cohen’s dz, calculated as the mean difference divided by the standard deviation of the difference scores; |dz| values of 0.2, 0.5, and 0.8 indicate small, medium, and large effects, respectively. Abbreviations: BMR: Basal Metabolic Rate; WHR: Waist-to-Hip Ratio; BFR: Body fat ratio; VFA: Visceral Fat Area; LUL-BF: Left Upper Limb Body Fat Percentage; RUL-BF: Right Upper Limb Body Fat Percentage; T-BF: Trunk Body Fat Percentage; LLL-BF: Left Lower Limb Body Fat Percentage; RLL-BF: Right Lower Limb Body Fat Percentage; LUL-M: Left Upper Limb Mass Percentage; RUL-M: Right Upper Limb Mass Percentage; T-M: Trunk Mass Percentage; LLL-M: Left Lower Limb Mass Percentage; RLL-M: Right Lower Limb Mass Percentage; TBW: Total Body Water; ECW: Extracellular Water; ICW: Intracellular Water; EI-ECW: Edema Index (ECW-based); EI-ECF: Edema Index (ECF-based). Obesity degree = (Current weight / Target weight) × 100%.

### 3.2 Longitudinal trajectories of body composition metrics by sex and predictive modeling of changes

Before interpreting the observed changes in fat and lean mass, we first examined whether fluid shifts during the intervention could have confounded the BIA estimates. As shown in Table 2, total body water, extracellular water, and intracellular water all exhibited significant decreases over the 4-week period (all *p* < 0.001), reflecting the expected physiological fluid loss accompanying glycogen depletion during early caloric restriction. Importantly, the edema indices (EI-ECW and EI-ECF) showed no significant changes throughout the intervention, indicating that water loss occurred proportionally across intracellular and extracellular compartments rather than reflecting pathological dehydration or fluid retention. These findings support the validity of interpreting the observed changes in fat and lean mass as genuine compositional shifts rather than artifacts of hydration changes, while also suggesting that a portion of the lean mass reduction detected by BIA may be explained in part by glycogen-associated water loss rather than representing equivalent structural muscle protein loss.

**Table 2:** Changes in body composition and metabolic parameters before and after the 4-week 5:2 fasting regimen, stratified by sex.

Characteristics	Sex	Before intervention	After intervention	Diff_CI	*P*-value (FDR)	Cohen’s *dz*
Weight (kg)	Male	102.89 ± 20.28	98.48 ± 20.16	−4.41 (−5.15, −3.67)	<0.001	−2.314
	Female	75.40 ± 11.20	73.12 ± 11.00	−2.28 (−2.61, −1.96)	<0.001	−1.562
BMI (kg/m^2^)	Male	33.21 ± 5.51	31.78 ± 5.46	−1.43 (−1.68, −1.18)	<0.001	−2.24
	Female	28.93 ± 3.79	28.05 ± 3.70	−0.88 (−1.01, −0.75)	<0.001	−1.519
BMR (kcal)	Male	1787.54 ± 183.66	1763.82 ± 184.02	−23.71 (−36.08, −11.35)	<0.001	−0.744
	Female	1350.07 ± 104.45	1337.14 ± 103.12	−12.94 (−18.65, −7.23)	<0.001	−0.501
Waist (cm)	Male	114.46 ± 16.94	109.64 ± 17.06	−4.83 (−5.81, −3.85)	<0.001	−1.908
	Female	96.25 ± 9.56	94.11 ± 9.86	−2.20 (−2.65, −1.75)	<0.001	−0.979
Hip (cm)	Male	112.40 ± 8.74	110.46 ± 8.78	−1.93 (−2.35, −1.52)	<0.001	−1.809
	Female	102.63 ± 6.28	101.30 ± 6.01	−1.33 (−1.56, −1.09)	<0.001	−1.27
WHR	Male	1.01 ± 0.08	0.98 ± 0.09	−0.03 (−0.03, −0.02)	<0.001	−1.168
	Female	0.94 ± 0.05	0.93 ± 0.05	−0.01 (−0.02, −0.01)	<0.001	−0.425
BFR (%)	Male	35.27 ± 6.12	33.44 ± 6.38	−1.83 (−2.34, −1.33)	<0.001	−1.411
	Female	39.31 ± 4.87	38.24 ± 4.78	−1.07 (−1.35, −0.79)	<0.001	−0.843
VFA (cm^2^)	Male	166.84 ± 60.13	150.36 ± 60.02	−16.10 (−20.40, −11.50)	<0.001	−1.746
	Female	145.98 ± 37.77	137.12 ± 38.69	−8.86 (−10.60, −7.13)	<0.001	−1.13
LUL-BF	Male	3.49 ± 2.24	3.01 ± 2.01	−0.45 (−0.55, −0.35)	<0.001	−1.379
	Female	2.46 ± 0.93	2.27 ± 0.87	−0.20 (−0.25, −0.20)	<0.001	−1.195
LUL/Normal-BF ratio %	Male	562.47 ± 339.18	485.37 ± 305.50	−70.83 (−90.50, −54.00)	<0.001	−1.431
	Female	267.23 ± 100.03	246.53 ± 92.32	−19.80 (−23.20, −16.75)	<0.001	−1.242
RUL-BF	Male	3.46 ± 2.25	2.98 ± 2.02	−0.45 (−0.60, −0.35)	<0.001	−1.473
	Female	2.44 ± 0.94	2.24 ± 0.87	−0.20 (−0.25, −0.15)	<0.001	−1.186
RUL/Normal BF ratio %	Male	558.07 ± 340.44	480.14 ± 305.38	−74.20 (−92.35, −53.95)	<0.001	−1.498
	Female	264.66 ± 100.44	243.72 ± 92.62	−20.05 (−23.55, −16.85)	<0.001	−1.242
T-BF	Male	19.05 ± 5.87	17.41 ± 5.95	−1.64 (−1.94, −1.34)	<0.001	−2.105
	Female	15.03 ± 3.50	14.23 ± 3.54	−0.80 (−0.94, −0.66)	<0.001	−1.306
T/Normal BF ratio %	Male	441.02 ± 126.68	402.35 ± 127.44	−38.67 (−46.03, −31.31)	<0.001	−2.038
	Female	292.07 ± 65.28	276.44 ± 66.77	−15.62 (−18.24, −13.01)	<0.001	−1.322
LLL-BF	Male	4.86 ± 1.48	4.55 ± 1.49	−0.35 (−0.45, −0.30)	<0.001	−1.233
	Female	4.41 ± 1.17	4.18 ± 1.05	−0.25 (−0.30, −0.20)	<0.001	−0.782
LLL/Normal BF ratio %	Male	276.05 ± 76.85	257.33 ± 77.48	−18.72 (−24.03, −13.41)	<0.001	−1.366
	Female	188.50 ± 49.71	178.42 ± 43.01	−9.05 (−11.40, −6.75)	<0.001	−0.791
RLL-BF	Male	4.92 ± 1.51	4.59 ± 1.50	−0.33 (−0.42, −0.24)	<0.001	−1.390
	Female	4.43 ± 1.18	4.20 ± 1.05	−0.25 (−0.30, −0.20)	<0.001	−0.787
RLL/Normal BF ratio %	Male	279.31 ± 78.17	260.22 ± 78.60	−19.09 (−24.53, −13.65)	<0.001	−1.361
	Female	189.66 ± 50.37	179.36 ± 43.36	−9.30 (−11.55, −6.95)	<0.001	−0.788
Lean mass	Male	61.84 ± 7.99	60.78 ± 8.00	−1.06 (−1.59, −0.52)	<0.001	−0.767
	Female	42.67 ± 4.55	42.10 ± 4.49	−0.57 (−0.82, −0.32)	<0.001	−0.505
LUL-M	Male	3.80 ± 0.66	3.68 ± 0.65	−0.15 (−0.20, −0.10)	<0.001	−0.865
	Female	2.35 ± 0.37	2.29 ± 0.37	−0.10 (−0.10, −0.05)	<0.001	−0.476
LUL/Normal M ratio %	Male	103.09 ± 10.57	101.00 ± 10.54	−2.09 (−3.40, −0.77)	0.004	−0.617
	Female	105.77 ± 12.10	104.21 ± 12.27	−1.56 (−2.54, −0.58)	0.003	−0.352
RUL-M	Male	3.83 ± 0.62	3.70 ± 0.63	−0.15 (−0.20, −0.15)	<0.001	−1.058
	Female	2.39 ± 0.36	2.33 ± 0.36	−0.10 (−0.15, −0.10)	<0.001	−0.603
RUL/Normal M ratio %	Male	103.75 ± 10.66	101.84 ± 10.10	−1.70 (−2.70, −0.55)	0.003	−0.601
	Female	107.10 ± 12.44	105.70 ± 12.53	−1.40 (−2.32, −0.47)	0.004	−0.333
T-M	Male	29.41 ± 3.84	28.66 ± 3.83	−0.75 (−0.90, −0.50)	<0.001	−1.149
	Female	20.58 ± 2.25	20.22 ± 2.27	−0.36 (−0.48, −0.24)	<0.001	−0.667
T/Normal M ratio %	Male	100.37 ± 6.54	99.25 ± 6.20	−1.00 (−1.80, −0.30)	0.01	−0.559
	Female	102.44 ± 5.73	101.71 ± 5.85	−0.73 (−1.26, −0.20)	0.009	−0.306
LLL-M	Male	10.03 ± 1.35	9.94 ± 1.33	−0.08 (−0.18, 0.01)	0.096	−0.335
	Female	6.87 ± 0.87	6.78 ± 0.87	−0.15 (−0.15, −0.05)	<0.001	−0.505
LLL/Normal M ratio %	Male	97.76 ± 5.41	98.44 ± 5.66	0.68 (−0.11, 1.46)	0.096	0.335
	Female	97.47 ± 5.20	97.32 ± 5.25	−0.15 (−0.67, 0.36)	0.595	−0.065
RLL-M	Male	10.03 ± 1.31	9.97 ± 1.31	−0.05 (−0.15, 0.05)	0.32	−0.201
	Female	6.89 ± 0.87	6.81 ± 0.87	−0.10 (−0.15, −0.05)	<0.001	−0.398
RLL/Normal M ratio %	Male	97.81 ± 5.61	98.80 ± 5.84	0.90 (0.20, 1.70)	0.019	0.451
	Female	97.65 ± 5.36	97.58 ± 5.40	−0.07 (−0.62, 0.48)	0.804	−0.028
TBW	Male	48.08 ± 6.21	47.25 ± 6.24	−0.82 (−1.25, −0.39)	<0.001	−0.742
	Female	33.21 ± 3.53	32.77 ± 3.49	−0.44 (−0.63, −0.25)	<0.001	−0.5
ECW	Male	18.06 ± 2.38	17.76 ± 2.39	−0.30 (−0.50, −0.10)	0.005	−0.586
	Female	12.59 ± 1.34	12.42 ± 1.32	−0.17 (−0.26, −0.08)	<0.001	−0.428
ICW	Male	30.00 ± 3.86	29.50 ± 3.85	−0.50 (−0.75, −0.26)	<0.001	−0.791
	Female	20.62 ± 2.21	20.35 ± 2.18	−0.28 (−0.39, −0.16)	<0.001	−0.532
EI-ECW	Male	0.33 ± 0.00	0.33 ± 0.00	0.00 (−0.00, 0.00)	0.757	−0.048
	Female	0.33 ± 0.00	0.33 ± 0.00	−0.00 (−0.00, 0.00)	0.804	−0.028
EI-ECF	Male	0.38 ± 0.00	0.38 ± 0.00	0.00 (−0.00, 0.00)	0.66	0.000
	Female	0.38 ± 0.00	0.38 ± 0.00	−0.00 (−0.00, 0.00)	0.656	−0.055
Isalt	Male	4.58 ± 0.65	4.53 ± 0.64	−0.10 (−0.15, −0.05)	0.02	−0.503
	Female	3.25 ± 0.37	3.21 ± 0.35	−0.10 (−0.10, −0.00)	<0.001	−0.371
Protein	Male	12.97 ± 1.68	12.75 ± 1.66	−0.22 (−0.32, −0.11)	<0.001	−0.804
	Female	8.91 ± 0.95	8.80 ± 0.95	−0.15 (−0.20, −0.10)	<0.001	−0.496

Data are presented as mean ± SD. Diff_CI = post-intervention (week 4) − pre-intervention (baseline). *P*-value (FDR) indicate adjustments for multiple comparisons using the Benjamini–Hochberg false discovery rate method. Effect size is Cohen’s d, calculated as the mean difference divided by the standard deviation of the differences, with |*d*z| values of 0.2, 0.5, and 0.8 indicate small, medium, and large effects, respectively. BMR: Basal Metabolic Rate; WHR: Waist-to-Hip Ratio; BFR: Body fat ratio; VFA: Visceral Fat Area; LUL-BF: Left Upper Limb Body Fat Percentage; RUL-BF: Right Upper Limb Body Fat Percentage; T-BF: Trunk Body Fat Percentage; LLL-BF: Left Lower Limb Body Fat Percentage; RLL-BF: Right Lower Limb Body Fat Percentage; LUL-M: Left Upper Limb Mass Percentage; RUL-M: Right Upper Limb Mass Percentage; T-M: Trunk Mass Percentage; LLL-M: Left Lower Limb Mass Percentage; RLL-M: Right Lower Limb Mass Percentage; TBW: Total Body Water; ECW: Extracellular Water; ICW: Intracellular Water; EI-ECW: Edema Index (ECW-based); EI-ECF: Edema Index (ECF-based).

**Figure 1:**
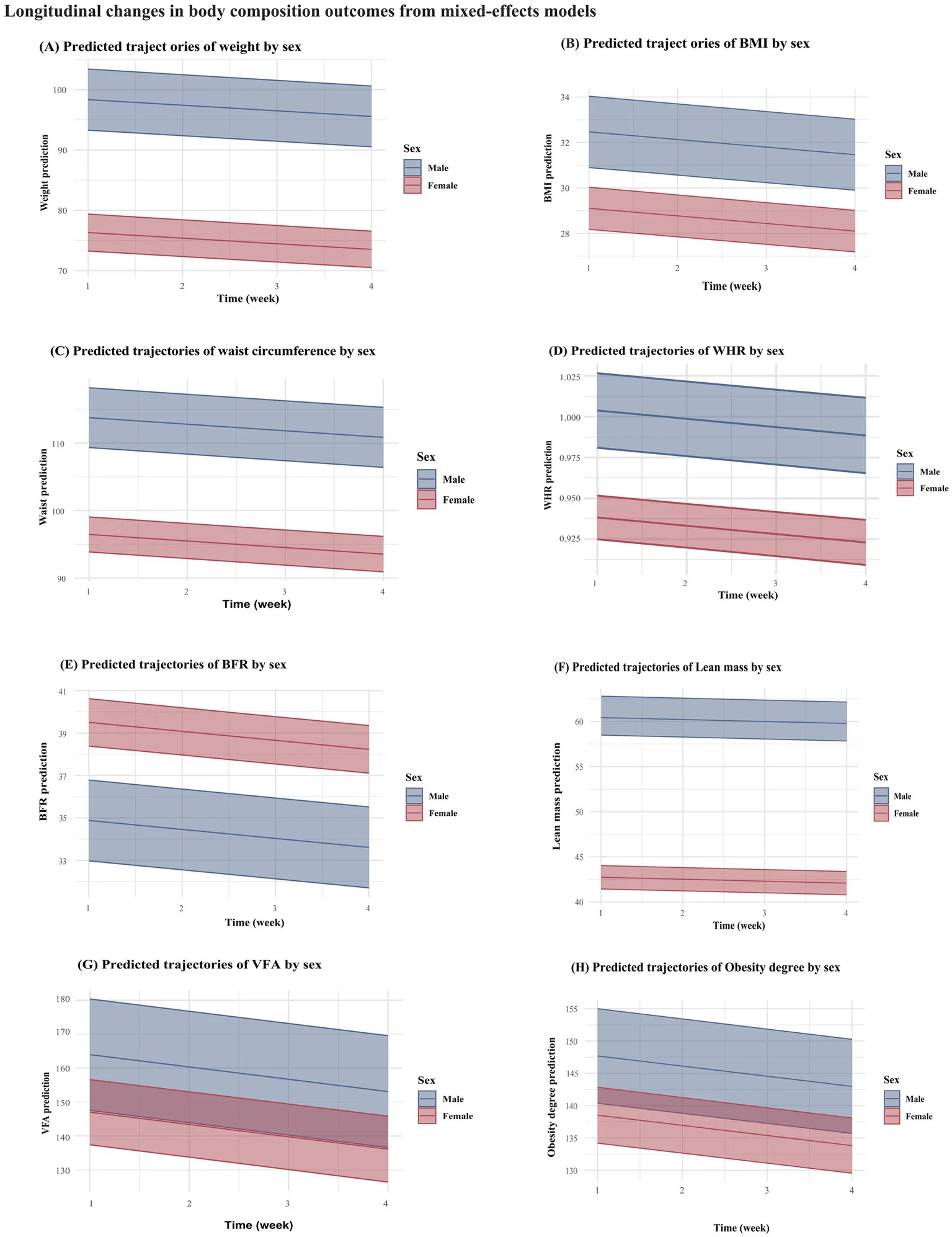
Longitudinal changes in body composition outcomes from mixed-effects models. **(A)** Predicted traject ories of weight by sex; **(B)** Predicted traject ories of body mass index (BMI) by sex; **(C)** Predicted trajectories of waist circumference by sex; **(D)** Predicted trajectories of waist-hip-ratio (WHR) by sex; **(E)** Predicted trajectories of body fat percentage (BFR) by sex; **(F)** Predicted trajectories of Lean mass by sex; **(G)** Predicted trajectories of visceral fat area (VFA) by sex; **(H)** Predicted trajectories of Obesity degree by sex. Obesity Degree (calculated as: Body Weight / Target Weight * 100%).

Having characterized the fluid dynamics, we next examined the temporal evolution of body composition metrics over the 4-week intervention. Visual inspection of the longitudinal trajectories (Supplementary Figure S1) suggested that metrics belonging to the fat compartment (body fat percentage, visceral fat area, and segmental fat mass), as well as derived anthropometric indices (body weight, BMI, waist circumference, WHR, and obesity degree), followed approximately linear decreasing patterns over time. For lean compartment metrics, however, the patterns were not uniform. Total lean mass and upper-body and trunk segmental lean mass (LUL-M, RUL-M, T-M) showed modest but statistically significant reductions in paired comparisons (Table 2), whereas lower-limb lean mass (LLL-M, RLL-M) and its ratios to normal reference values showed no significant changes (all *p* > 0.05), suggesting that the lean mass reduction was not uniformly distributed across body regions. In contrast, body water, protein, and mineral content showed no appreciable trends beyond the expected decreases in water compartments noted above. Given the linear changes in fat and lean tissue metrics, linear mixed-effects models were employed to predict the rate of change for each metric over time. As depicted in Figure 1 and Supplementary Table S2, following the implementation of the 5:2 fasting regimen, both males and females exhibited significant decreasing trends in body weight, BMI, BMR, waist circumference, WHR, BFR, VFA, obesity degree, and lean mass over time (all *p* < 0.05). These results suggest that while the 5:2 fasting regimen was associated with reduced body weight and fat content, attention should be paid to the concurrent loss of muscle mass warrants attention.

### 3.3 Association between weight change and body composition stratified by sex

**Figure 2:**
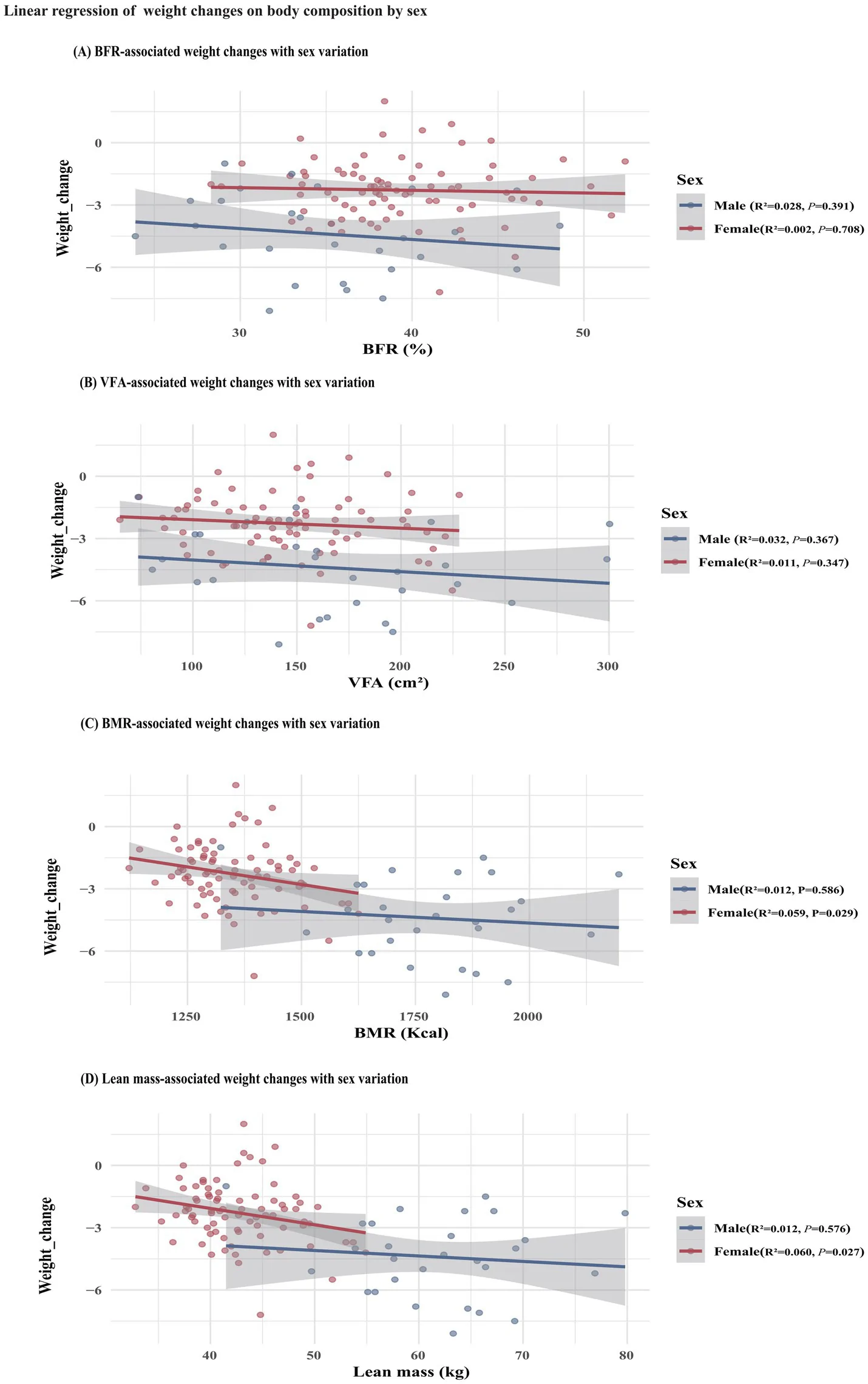
Linear regression of weight changes on body composition by sex. **(A)** Body fat percentage (BFR)-associated weight changes with sex variation; **(B)** Visceral fat area (VFA)-associated weight changes with sex variation; **(C)** Basal Metabolic Rate (BMR)-associated weight changes with sex variation; **(D)** Lean mass-associated weight changes with sex variation.

To investigate whether weight loss was associated with initial fat mass and muscle mass in males and females, Pearson correlation analysis was performed (Figure 2). In females, weight loss correlated significantly with initial muscle mass and BMR, but not with initial fat mass. In contrast, male weight loss showed no significant correlation with either initial muscle mass or initial fat mass. Additionally, we analyzed the associations of age on changes in weight, fat mass, and muscle mass. As illustrated in Supplementary Figure S2, under the 4-week time-restricted energy intervention, correlations between age and changes in body weight, waist-to-hip ratio, visceral fat area, basal energy expenditure (BMR), and lean tissue mass were weak in both sexes. The coefficients of determination (*R*^2^) were all below 0.100 and statistically non-significant. Notably, the *R*^2^ values for the significant correlations in females (approximately 0.06) indicate that baseline muscle mass and BMR explained only a small proportion (about 6%) of the variance in weight loss, suggesting limited predictive utility.

### 3.4 Cluster analysis of body composition changes by sex and association with metric variations

**Figure 3:**
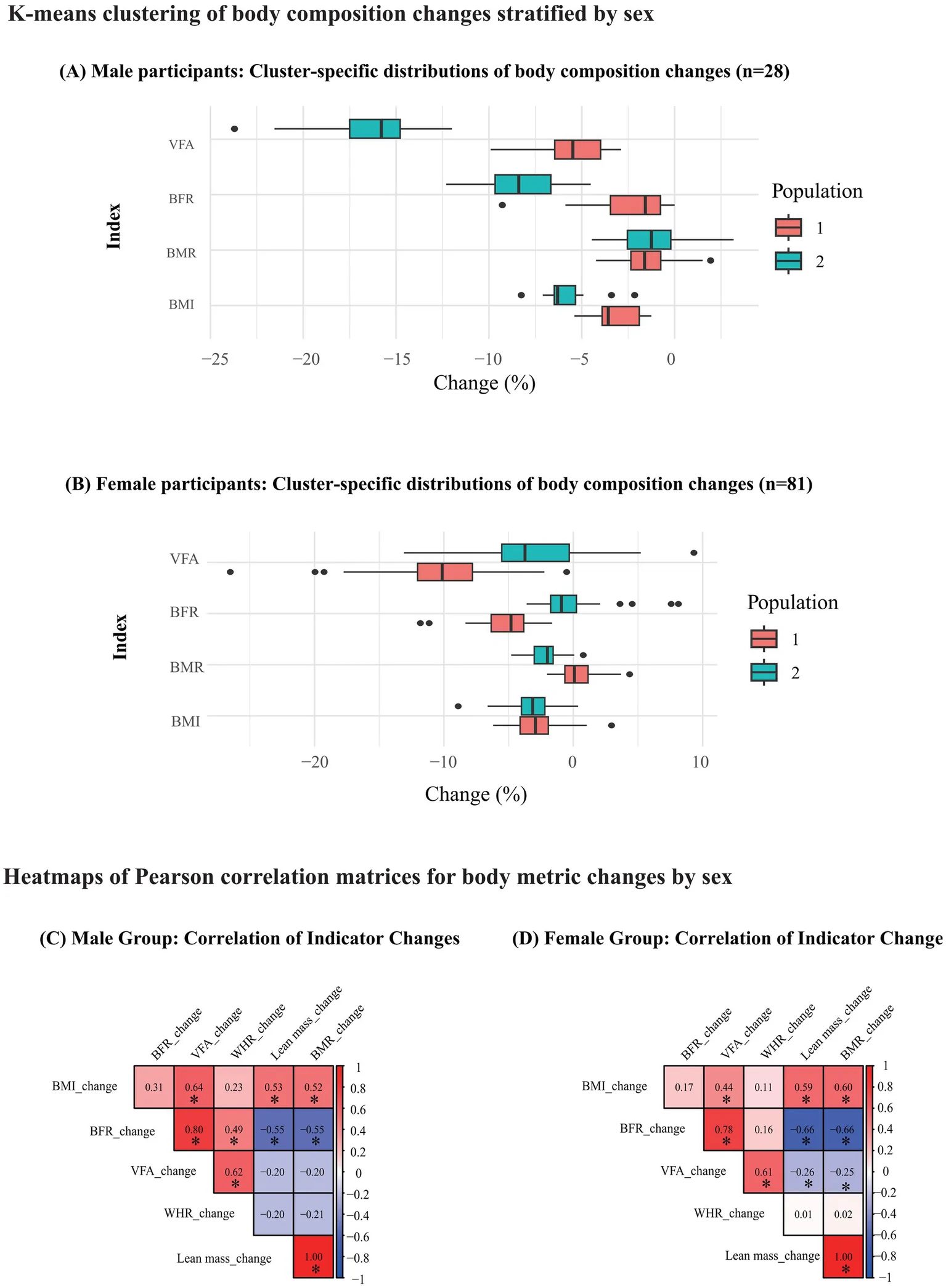
K-means clustering of body composition changes stratified by sex. **(A)** Male participants: Cluster-specific distributions of body composition changes (*n* = 28); **(B)** Female participants: Cluster-specific distributions of body composition changes (*n* = 81); **(C)** Male Group: Correlation of Indicator Change; **(D)** Female Group: Correlation of Indicator Changes.

To further examine the association between muscle mass and basal metabolic rate (BMR) on weight and fat loss in females, K-means cluster analysis was conducted (Figure 3). The stability of these cluster assignments was confirmed by Bootstrap resampling validation (mean Jaccard index: 0.900 for males, 0.816 for females; Supplementary Table S3). Female participants clustered into two distinct groups. The group with higher baseline BMR exhibited greater changes in weight and fat loss, whereas no such clustering based on BMR level was observed in males. Finally, correlation analysis of metric changes, visualized via a heatmap, revealed a strong positive correlation between muscle mass and BMR, suggesting that higher baseline muscle mass is associated with BMR. Conversely, both muscle mass and BMR showed inverse correlations with changes in body fat percentage (BFR). This finding indicate that while the time-restricted energy intervention was associated with reductions in overall BMI and fat, it may also be accompanied by reductions in muscle mass and basal metabolic rate.

## 4 Discussion

The 5:2 intermittent fasting regimen, characterized by ad libitum eating for 5 days per week and significant caloric restriction (~500–600 kcal) on two non-consecutive days, has gained prominence as a weight management and metabolic health strategy (27). Our retrospective analysis of serial body composition data during this 4-week intervention revealed consistent, time-dependent reductions in body weight, BFR, and VFA across both sexes.

BFR serves as a critical indicator of adiposity, and its reduction is associated with improved body composition and metabolic health (28, 29). VAT localized within the abdominal cavity, constitutes a metabolically active depot strongly linked to insulin resistance and cardiovascular risk (29). The observed VAT reduction aligns with enhanced insulin sensitivity and attenuated cardiometabolic risk. These findings not only corroborate prior studies demonstrating the weight-loss efficacy of 5:2 fasting (10, 30–32) but further reveal a linear controlled trajectory of weight and fat loss. This regulated progression was associated with several physiological features, including a relatively modest reduction in lean mass compared with rapid weight-loss regimens, as well as preservation of metabolic adaptation, and favorable patterns of behavioral adherence and dietary restructuring (33–36).

Our data revealed a modest but statistically significant decline in lean mass over the 4-week intervention in both sexes. Several methodological considerations should be taken into account when interpreting this change. BIA estimates lean mass indirectly based on total body water and electrical conductivity (37), and thus cannot distinguish between skeletal muscle, glycogen, and other fat-free components. Previous studies have shown that the early phase of caloric restriction is accompanied by glycogen depletion and associated water loss (approximately 3–4 grams of water per gram of glycogen) (38–40). Therefore, the reduction in lean mass detected in our study may be partially attributable to this physiological process. Our data support this interpretation: total body water, as well as intracellular and extracellular water, all decreased significantly, whereas the edema indices (EI-ECW and EI-ECF) showed no significant changes throughout the intervention, indicating that water loss occurred proportionally across compartments—consistent with a physiological fluid shift in response to energy restriction rather than pathological dehydration or fluid retention. However, the magnitude of total body water reduction was not fully proportional to the magnitude of lean mass loss, suggesting that a certain degree of structural skeletal muscle protein catabolism may still have occurred. Future studies employing criterion methods such as dual-energy X-ray absorptiometry (DXA) or computed tomography (CT) will be helpful to further clarify this issue (41). Nevertheless, the modest trend toward muscle mass reduction warrants attention. Muscle is essential for maintaining metabolic rate, physical strength, and overall health. While our study documented changes only within the 4-week timeframe, prolonged energy restriction or suboptimal dietary patterns may be associated with exacerbated muscle catabolism (36, 42). Consequently, preserving lean mass during weight loss is paramount. Strategic protein intake and resistance training are recommended to mitigate muscle loss risks (43, 44).

Sex is significantly associated with differences in body composition responses. Physiological and hormonal differences typically render females more susceptible to adiposity (particularly gluteofemoral fat deposition) and lower baseline muscle mass than males (45, 46). Our data indicate that muscle mass and BMR are significantly associated with weight loss dynamics, especially in females. The prevalent high-adiposity/low-muscle phenotype in women suggests that higher baseline lean mass is correlated with higher BMR, and this association may be relevant to more favorable weight loss responses. Interventions combining dietary management with physical activity that aim to augment muscle mass may be particular relevance for females (47–49). In males, inherently higher testosterone levels are associated with muscle anabolism and lipid oxidation (50). Consequently, energy intake regulation appeared to be more strongly associated with weight loss velocity than muscle-centric mechanisms in this study (51, 52). Consistent with this observation, the decline in BMR over the 4-week intervention is consistent with the expected metabolic adaptation to energy deficit, and should not be interpreted as BMR decline driving reduced weight- loss efficiency. Rather, the association between baseline BMR and weight loss outcomes should be understood as correlational.

Several considerations regarding the observed associations warrant attention. Although baseline muscle mass and BMR were significantly correlated with weight loss in females, the *R*^2^ values of approximately 0.06 indicate that these factors accounted for only about 6% of the inter-individual variance in weight loss. This modest effect size suggests that the clinical predictive value of baseline muscle mass or BMR alone is limited. The majority of weight loss variability is likely explained by other determinants, including energy intake adherence, physical activity levels, baseline adiposity, and individual differences in metabolic adaptation. Therefore, while the associations were statistically significant, they should not be interpreted as strong predictors of weight loss success. Rather, these findings are hypothesis-generating and suggest that muscle-centric strategies may play a small but potentially modulatory role in female weight loss responses. Future adequately powered interventional studies are needed to determine whether targeting muscle mass or BMR translates into clinically meaningful improvements in weight loss outcomes. In addition, the BMR-based subgroup identification in the clustering analysis should be interpreted with caution, as BMR is highly correlated with muscle mass and overall body size (53, 54). The observation that the high-BMR group achieved greater weight loss may partly reflect greater baseline muscle mass and body size, rather than representing a distinct metabolic phenotype. This interpretation does not diminish the hypothesis-generating value of the clustering results, but it underscores that the observed patterns should not be interpreted as evidence for a biologically distinct subgroup.

Importantly, this study was observational and retrospective in nature, and therefore all reported associations should be interpreted as correlational rather than causal. While we observed significant associations between baseline muscle mass, BMR, and weight loss outcomes in females, these findings do not imply that increasing muscle mass or BMR directly causes greater weight loss. Unmeasured confounding variables (e.g., habitual physical activity, dietary adherence, hormonal status) may have contributed to these associations (55–57). Thus, all interpretations should be viewed as hypothesis-generating and warrant confirmation in prospective, interventional studies.

Age exerted no significant effect on body composition changes in our study, likely attributable to metabolic homogeneity within our middle-aged participants and the absence of elderly individuals or confounding chronic medications. Importantly, our findings should not be extrapolated to older populations, as aging is known to be associated with progressive declines in basal metabolic rate and muscle mass—a process further exacerbated by malnutrition and physical inactivity in the elderly (58–60). Future weight management strategies must incorporate age-specific adaptations to address these vulnerabilities.

### 4.1 Limitations and future directions

Several limitations of this study warrant acknowledgment. First, the exclusive enrollment of adults (18–60 years) precludes generalization to pediatric or elderly populations, necessitating age-stratified validation. Second, the absence of serum metabolic profiling (e.g., insulin, lipidomics, inflammatory cytokines) obscures the intervention’s impact on systemic metabolic pathways. Third, the 4-week duration fails to capture long-term trajectories, including weight-loss plateau or rebound dynamics. Fourth, the single-arm design without a control group precludes attributing the observed changes specifically to the 5:2 regimen, as we cannot exclude that these effects reflect general caloric restriction rather than regimen-specific mechanisms. However, this study was not designed to establish superiority over other dietary approaches, but rather to provide high-resolution, dynamic characterization of short-term body composition trajectories—a question that remains valid within a single-arm design. Fifth, the intervention included multiple lifestyle components (exercise, behavioral counseling, meal-timing strategies, and dietary education) in addition to the 5:2 fasting protocol. Adherence to these components was not objectively monitored, and quantitative data on actual calorie intake, macronutrient compliance, and physical activity levels were not collected. Therefore, the observed effects cannot be attributed exclusively to the fasting regimen. Sixth, the small number of male participants (*n* = 28) may have limited statistical power to detect modest sex-specific associations, and male-specific findings should be considered preliminary. Seventh, phase angle (PhA) was not available due to device limitations, precluding direct distinction between true muscle loss and hydration shifts; however, our body water compartment analysis partially mitigates this concern.

Importantly, the observed associations between baseline muscle mass, BMR, and weight loss in females are correlational and do not establish causation. The low *R*^2^ values (≈0.06) further indicate limited predictive value. Thus, all sex-specific recommendations should be interpreted as hypothesis-generating rather than clinically directive.

Future studies should:(1) Expand cohorts to include age-extreme groups for metabolic response analysis; (2) Employ multi-omics approaches (metabolomics/proteomics) to decipher molecular mechanisms; (3) Extend follow-up to ≥12 weeks to quantify weight-loss maintenance rates; (4) Conduct randomized trials comparing efficacy-safety profiles across dietary regimens (e.g., 5:2 fasting vs. time-restricted eating vs. continuous caloric restriction).

## 5 Conclusion

Within the 4-week 5:2 fasting intervention, both adult males and females exhibited consistent linear reductions in body weight, body fat percentage, and visceral adipose tissue. However, a modest trend toward lean mass loss was concurrently observed. Baseline muscle mass and BMR were significant correlates of weight loss in females, suggesting that muscle-centric strategies may be particularly relevant for this subgroup. In males, weight loss appeared to be primarily associated with energy intake regulation rather than baseline body composition. These findings are observational and hypothesis-generating; causality cannot be inferred from the present data. Future prospective studies are warranted to validate these sex-specific associations.

## Data Availability

The original contributions presented in the study are included in the article/Supplementary material, further inquiries can be directed to the corresponding author.

## Supplementary material

Supplementary Figure S1 Individual Trajectories of Body Composition Indicators During One-Month 5:2 Intermittent Fasting. **(A)** Body Morphology: Weight, body mass index (BMI), Visceral fat area (VFA), Waist circumference, Hip, Waist-hip-ratio (WHR); **(B)** Body Water and Solid Composition Balance Indicators: Cell number, Protein, Isalt, Total Body Water (TBW), Extracellular Water (ECW), Intracellular Water (ICW); **(C)** Total Fat and Segmental Fat: Body fat percentage (BFR), LUL/Normal-BF ratio %, RUL/Normal-BF ratio %, T/Normal-BF ratio %, LLL/Normal-BF ratio %, RLL/Normal-BF ratio %; **(D)** Muscle Mass and Segmental Muscle: Lean mass, Left Upper Limb Mass (LUL-M), Right Upper Limb Mass (RUL-M), Trunk Mass (T-M), Left Lower Limb Mass (LLL-M), Right Lower Limb Mass (RLL-M); **(E)** Body Composition Control: Edema Index (ECW-based) (EI-ECW), Edema Index (ECF-based) (EI-ECF). (Assuming ECF refers to Extracellular Fluid), Fat Control, Muscle Control, Body Weight Control. The control variable is equal to the target value minus the actual value.

Supplementary Figure S2 Linear regression of body composition changes on age by sex. **(A)** Age-associated body mass index (BMI) changes with sex variation; **(B)** Age-associated waist-hip-ratio (WHR) changes with sex variation; **(C)** Age-associated body fat percentage (BFR) changes with sex variation; **(D)** Age-associated Visceral fat area (VFA) changes with sex variation; **(E)** Age-associated Lean mass changes with sex variation.
