# Supplementary material for: Temporal changes in physiological parameters in Chinese adults during a month-long 5:2 fasting regimen: an exploratory observational study
Source: Front Nutr. 2026 Sep 7;13:1866854. doi: 10.3389/fnut.2026.1866854 (PMC13592090; doi:10.3389/fnut.2026.1866854)

Linear regression of body composition changes on age by sex

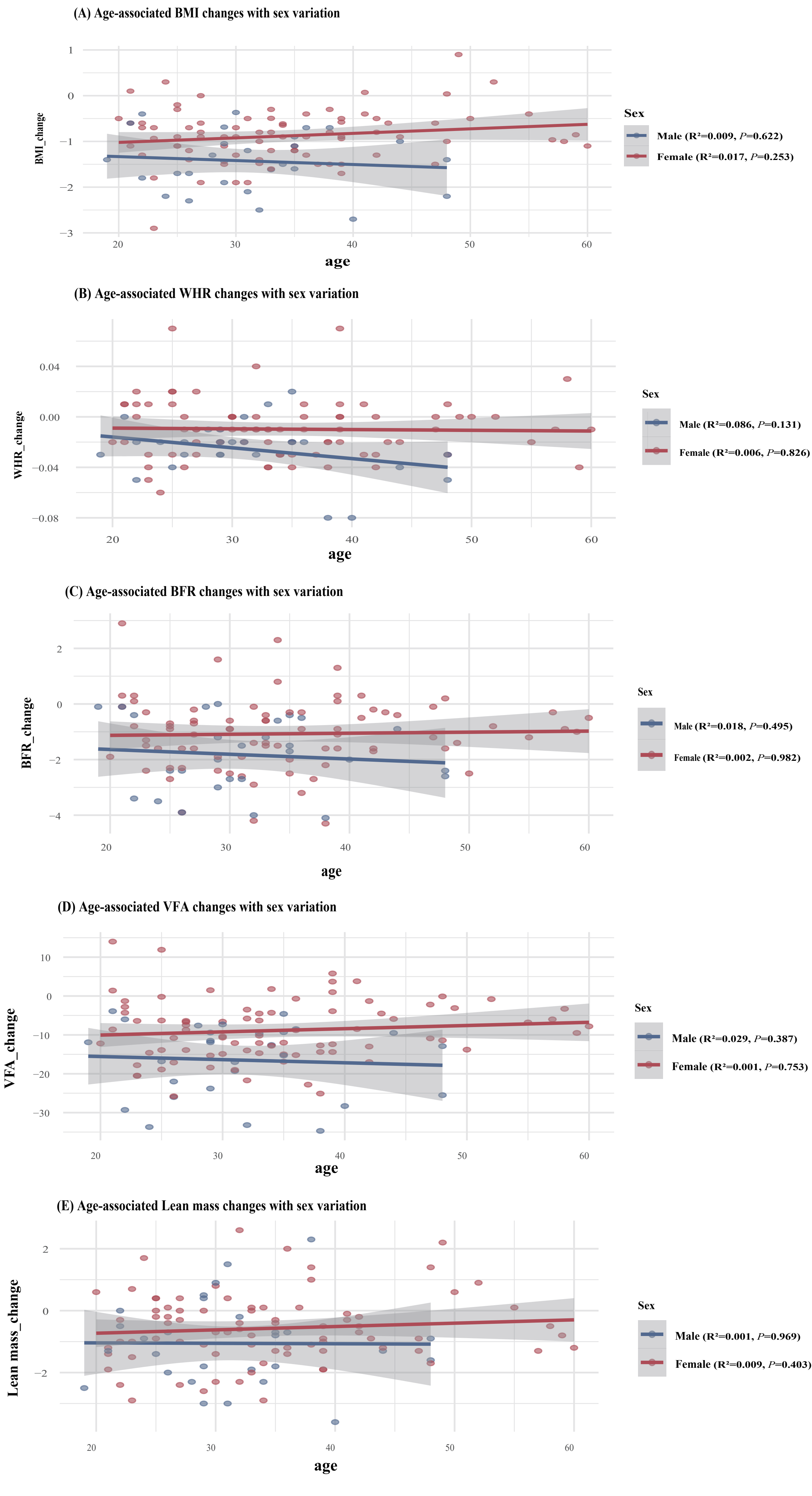

Supplement: Supplementary file 2 [file Image_2.pdf]
