# Supplementary material for: Temporal changes in physiological parameters in Chinese adults during a month-long 5:2 fasting regimen: an exploratory observational study
Source: Front Nutr. 2026 Sep 7;13:1866854. doi: 10.3389/fnut.2026.1866854 (PMC13592090; doi:10.3389/fnut.2026.1866854)
Supplement: Supplementary file 3 [file Table_1.DOCX]

Table S1. Baseline characteristics of participants meeting age criteria (screened population) compared with those finally included in the analysis

| Characteristics | Sex | Screened population | Finally population | Diff_CI | *P-value (FDR)* | *Cohen's dz* |
| --- | --- | --- | --- | --- | --- | --- |
| Age | Male | 32.36 ± 8.07 | 31.39 ± 7.52 | -1.00 (-4.00, 2.00) | 0.91 | -0.121 |
|  | Female | 33.73 ± 9.31 | 34.44 ± 9.87 | 0.00 (-2.00, 3.00) | 0.91 | 0.076 |
| Weight (kg) | Male | 95.21 ± 16.68 | 102.89 ± 20.28 | 8.00 (0.10, 15.30) | 0.206 | 0.444 |
|  | Female | 76.44 ± 12.35 | 75.40 ± 11.20 | -0.30 (-3.20, 2.30) | 0.91 | -0.086 |
| BMI (kg/m^2^) | Male | 31.31 ± 4.56 | 33.21 ± 5.51 | 2.00 (-0.10, 4.00) | 0.206 | 0.401 |
|  | Female | 29.16 ± 4.03 | 28.93 ± 3.79 | -0.00 (-0.90, 0.90) | 1 | -0.059 |
| BMR (kcal) | Male | 1723.87 ± 174.17 | 1787.54 ± 183.66 | 73.00 (-0.00, 142.00) | 0.206 | 0.362 |
|  | Female | 1357.51 ± 123.08 | 1350.07 ± 104.45 | -3.00 (-30.00, 22.00) | 0.91 | -0.062 |
| Waist (cm) | Male | 108.38 ± 14.69 | 114.46 ± 16.94 | 6.30 (-0.10, 13.30) | 0.206 | 0.404 |
|  | Female | 96.49 ± 10.14 | 96.25 ± 9.56 | 0.00 (-2.30, 2.50) | 0.983 | -0.024 |
| Hip (cm) | Male | 109.23 ± 7.25 | 112.40 ± 8.74 | 3.50 (0.00, 6.70) | 0.206 | 0.422 |
|  | Female | 103.18 ± 6.62 | 102.63 ± 6.28 | -0.20 (-1.70, 1.20) | 0.91 | -0.084 |
| WHR | Male | 0.99 ± 0.08 | 1.01 ± 0.08 | 0.02 (-0.02, 0.05) | 0.745 | 0.209 |
|  | Female | 0.93 ± 0.05 | 0.94 ± 0.05 | 0.00 (-0.01, 0.02) | 0.91 | 0.058 |
| BFR (%) | Male | 33.47 ± 6.01 | 35.27 ± 6.12 | 1.80 (-0.66, 4.26) | 0.335 | 0.299 |
|  | Female | 39.69 ± 5.19 | 39.31 ± 4.87 | -0.37 (-1.65, 0.90) | 0.91 | -0.073 |
| VFA (cm^2^) | Male | 146.45 ± 53.23 | 166.84 ± 60.13 | 19.60 (-2.40, 45.70) | 0.207 | 0.375 |
|  | Female | 148.70 ± 39.39 | 145.98 ± 37.77 | -2.00 (-12.10, 7.70) | 0.91 | -0.07 |
| Lean mass | Male | 59.06 ± 7.57 | 61.84 ± 7.99 | 3.20 (-0.00, 6.20) | 0.206 | 0.363 |
|  | Female | 42.99 ± 5.37 | 42.67 ± 4.55 | -0.10 (-1.30, 1.00) | 0.91 | -0.062 |
| TBW | Male | 45.90 ± 5.90 | 48.08 ± 6.21 | 2.52 (-0.00, 4.90) | 0.206 | 0.365 |
|  | Female | 33.48 ± 4.19 | 33.21 ± 3.53 | -0.10 (-1.00, 0.70) | 0.91 | -0.065 |
| ECW | Male | 17.20 ± 2.26 | 18.06 ± 2.38 | 1.00 (0.00, 1.90) | 0.206 | 0.379 |
|  | Female | 12.72 ± 1.62 | 12.59 ± 1.34 | -0.10 (-0.40, 0.30) | 0.91 | -0.083 |
| ICW | Male | 28.70 ± 3.66 | 30.00 ± 3.86 | 1.50 (-0.00, 3.00) | 0.206 | 0.352 |
|  | Female | 20.76 ± 2.58 | 20.62 ± 2.21 | -0.00 (-0.60, 0.50) | 0.91 | -0.053 |
| Isalt | Male | 4.37 ± 0.61 | 4.58 ± 0.65 | 0.20 (-0.00, 0.50) | 0.207 | 0.343 |
|  | Female | 3.27 ± 0.42 | 3.25 ± 0.37 | -0.00 (-0.10, 0.10) | 0.91 | -0.052 |
| Protein | Male | 12.40 ± 1.58 | 12.97 ± 1.68 | 0.70 (-0.00, 1.30) | 0.206 | 0.355 |
|  | Female | 8.97 ± 1.11 | 8.91 ± 0.95 | -0.00 (-0.30, 0.20) | 0.91 | -0.054 |

The screened population comprised 417 individuals (141 males and 276 females) who met the initial age eligibility criteria (18–60 years) after excluding 235 participants aged <18 or >60 years from the initial 652 overweight/obese adults. The final included consisted of 109 participants (28 males and 81 females) who completed the 4-week intervention and attended all scheduled weekly follow-up visits. Data are presented as mean ± standard deviation (SD). Mean difference (Diff_CI) was calculated as the value for the final included participants minus that for the screened population. *P*-value (FDR) indicate adjustments for multiple comparisons using the Benjamini–Hochberg false discovery rate method. Effect size is Cohen's d, calculated as the mean difference divided by the standard deviation of the differences, with |dz| values of 0.2, 0.5, and 0.8 indicate small, medium, and large effects, respectively.No significant differences were observed between the two groups for any of the baseline characteristics (all *P*-value (FDR) > 0.05), indicating that participants who completed the 4-week intervention were comparable at baseline to those who met the initial eligibility criteria but were not included due to incomplete intervention or follow-up.

Abbreviations: BMR: Basal Metabolic Rate; WHR: Waist-to-Hip Ratio; BFR: Body fat ratio; VFA: Visceral Fat Area; TBW: Total Body Water; ECW: Extracellular Water; ICW: Intracellular Water.

TableS2. Mixed-effects models examining longitudinal changes in body composition indicators

| Parameter | Weight | BMI | Waist | WHR | BFR | Lean mass | VFA | Obesity degree |
| --- | --- | --- | --- | --- | --- | --- | --- | --- |
| **Fixed Effects** |  |  |  |  |  |  |  |  |
| Intercept | 113.52 (5.06)*** | 34.88 (1.57)*** | 122.81 (4.45)*** | 1.03 (0.02)*** | 39.18 (1.91)*** | 66.04 (2.03)*** | 202.64 (16.49)*** | 159.23 (7.33)*** |
| Time | -0.93 (0.06)*** | -0.33 (0.02)*** | -0.97 (0.08)*** | -0.005 (0.001)*** | -0.42 (0.04)*** | -0.22 (0.04)*** | -3.63 (0.27)*** | -1.57 (0.09)*** |
| Sex (Female) | -23.81 (2.98)*** | -3.36 (0.92)*** | -17.32 (2.62)*** | -0.07 (0.01)*** | 4.62 (1.13)*** | -18.35 (1.19)*** | -16.96 (9.71). | -9.17 (4.31)* |
| Age | -0.37 (0.14)** | -0.06 (0.04) | -0.24 (0.12). | -0.0005 (0.0006) | -0.12 (0.05)* | -0.14 (0.06)* | -1.04 (0.45)* | -0.36 (0.19). |
| **Random Effects** |  |  |  |  |  |  |  |  |
| σ² Intercept | 187.52 | 18.42 | 138.82 | 0.0036 | 25.72 | 29.24 | 1903.06 | 400.88 |
| σ² Time | 0.28 | 0.03 | 0.33 | 0.00002 | 0.05 | 0.05 | 3.85 | 0.70 |
| ρ (Int-Time) | -0.20 | -0.25 | 0.00 | 0.05 | -0.006 | -0.11 | 0.11 | -0.25 |
| σ² Residual | 0.42 | 0.05 | 1.56 | 0.00018 | 0.61 | 0.47 | 21.57 | 1.17 |
| **Model Info** |  |  |  |  |  |  |  |  |
| Observations | 436 | 436 | 436 | 436 | 436 | 436 | 436 | 436 |
| Participants | 109 | 109 | 109 | 109 | 109 | 109 | 109 | 109 |
| REML Criterion | 1823.1 | 905.0 | 2146.9 | -1955.1 | 1620.8 | 1558.2 | 3271.9 | 2229.3 |

Notes:

Values represent estimates (standard errors). Significance codes: ***p<0.001, **p<0.01, *p<0.05

All models included random intercepts and time slopes with participant ID (1 + time | ID)

Gender reference category is Male (Female coefficients represent difference from males)

Abbreviations: BFR: Body fat ratio; WHR: Waist-to-Hip Ratio; VFA: Visceral Fat Area

Table S3. Bootstrap validation of clustering stability by sex

| Group | Sample size (n) | Jaccard index (Cluster 1) | Jaccard index (Cluster 2) | Mean Jaccard index | Stability classification |
| --- | --- | --- | --- | --- | --- |
| Male | 28 | 0.903 | 0.897 | 0.900 | Highly stable (> 0.85) |
| Female | 81 | 0.829 | 0.803 | 0.816 | Moderately stable (0.70) |

Bootstrap resampling was performed with 1,000 iterations. The Jaccard similarity index measures the average proportion of samples that were assigned to the same cluster across resampled datasets. According to established conventions, Jaccard index > 0.85 indicates highly stable clustering, 0.70–0.85 indicates moderately stable clustering, and < 0.60 indicates unstable clustering. The mean Jaccard index for male clustering (0.900) exceeded the threshold for high stability, confirming that the cluster structure in males is robust despite the smaller sample size. Female clustering also demonstrated moderate-to-good stability (mean Jaccard = 0.816).
